# Impact of Mediterranean diet on metabolic syndrome, cancer and longevity

**DOI:** 10.18632/oncotarget.13553

**Published:** 2016-11-24

**Authors:** Nicola Di Daniele, Annalisa Noce, Maria Francesca Vidiri, Eleonora Moriconi, Giulia Marrone, Margherita Annicchiarico-Petruzzelli, Gabriele D’Urso, Manfredi Tesauro, Valentina Rovella, Antonino De Lorenzo

**Affiliations:** ^1^ Department of Systems Medicine, Hypertension and Nephrology Unit, University of Rome “Tor Vergata”, Italy; ^2^ Department of Biomedicine and Prevention, Division of Clinical Nutrition and Nutrigenomic, University of Rome “Tor Vergata”, Italy; ^3^ Biochemistry Laboratory, IDI-IRCCS, Department of Experimental Medicine and Surgery, University of Rome “Tor Vergata”, Italy

**Keywords:** Mediterranean diet, public health, obesity, cancer, antioxidant

## Abstract

Obesity symbolizes a major public health problem. Overweight and obesity are associated to the occurrence of the metabolic syndrome and to adipose tissue dysfunction. The adipose tissue is metabolically active and an endocrine organ, whose dysregulation causes a low-grade inflammatory state and ectopic fat depositions. The Mediterranean Diet represents a possible therapy for metabolic syndrome, preventing adiposopathy or “sick fat” formation.

The Mediterranean Diet exerts protective effects in elderly subjects with and without baseline of chronic diseases. Recent studies have demonstrated a relationship between cancer and obesity. In the US, diet represents amount 30-35% of death causes related to cancer. Currently, the cancer is the second cause of death after cardiovascular diseases worldwide. Furthermore, populations living in the Mediterranean area have a decreased incidence of cancer compared with populations living in Northern Europe or the US, likely due to healthier dietary habits. The bioactive food components have a potential preventive action on cancer. The aims of this review are to evaluate the impact of Mediterranean Diet on onset, progression and regression of metabolic syndrome, cancer and on longevity.

## INTRODUCTION

Obesity and metabolic syndrome (MetS) showed a significant increase in the last decades, becoming a public health problem due to high hospitalization rates, treatment costs and impaired quality of life.

Both obesity that MetS are characterized by insulin resistance that expose the patients to an increased risk of cardiovascular disease and cancer, despite the healthy nutritional habits [1]. In fact only 5-10% of cancer cases are linked to genetic causes while 90-95% can be attributed to environment and lifestyle [2].

The Mediterranean Diet (MD) is characterized by an adequately balanced combination of fruit and vegetables, fish, cereals, and polyunsaturated fats, with a reduced consumption of meat and dairy products and moderate intake of alcohol, primarily red wine. The value of this diet lies in its ability to preserve the state of health and improve longevity, as United Nations Educational, Scientific and Cultural Organization (UNESCO) declared in 2010 [3].

Scientific evidence has showed that the adoption of the Mediterranean diet is a protective factor against the onset of various types of cancer, cardiovascular disease, aging and obesity. As shown by the Epic (European Prospective Investigation into Cancer and Nutrition) study, the MD is the most effective in the prevention of several chronic diseases, including cancer.

The nutrients that are found in abundance in the Mediterranean diet have anti-cancer, anti-inflammatory, anti-obesity properties and contribute together to the maintenance of health status. The anti-tumor effects of the Mediterranean diet are mostly due to the combination of antioxidant elements, fiber and polyunsaturated fats. This dietary pattern is therefore essential as a preventive measure against the onset of cancer and other chronic diseases but also to reduce health care costs.

## MEDITERRANEAN DIET, METABOLIC SYNDROME (METS) AND OBESITY

### Definition and classification of Mets

Overweight and obesity are highly prevalent worldwide. One billion people in the world are overweight and 300 million are considered obese. Several studies estimate that in 2030, approximately 2 billion people will be overweight and 1,12 billion obese [4]. In the United States, abdominal obesity has tripled during the past 40 years [5]. More than 25% of the US population can be classified as having the MetS [6]. MetS prevalence has increased from 34.3% to 38.5% during the past 15 years [7, 8]. Given the prevalence of the phenomenon, obesity is considered an important public health problem, with a strong impact on health status, economy and quality of life. The accumulated adipose tissue, particularly central adiposity, generates a chronic low-grade inflammatory state that is the basis of metabolic disturbances, such as insulin resistance, dyslipidemia and hyperuricemia. Insulin resistance is the “core” of the MetS and abdominal obesity plays a central role in this syndrome.

MetS is characterized by dyslipidemia and arterial hypertension, which are associated with increased risk of type 2 diabetes mellitus (T2DM) and cardiovascular diseases (CVD) [9]. The relationship between obesity and T2DM is so strong that the new term “diabesity” has been introduced. It is a global pandemic favoured by an obesogenic environment that promotes consumption of energy-dense foods and discourages energy expenditure, with the result of weight gain. In recent years there has been an increase in the incidence of obesity in young people, with the result that T2DM and MetS are not exclusive to adulthood but are increasingly observed in childhood [10].

In addition, several types of cancers, including uterine, breast, colon, prostate and kidney are obesity driven [8, 9]. Studies indicate that, over the past decade, 25% of all cancers could be prevented by reducing the rate of obesity [13–15].

Several definitions for MetS have been proposed. In 1988, Reaven first spoke of “the syndrome X,” which is currently defined “Metabolic Syndrome”. Reaven noticed a close link between risk factors of CVD (glucose intolerance, hyperinsulinemia, high serum triglycerides, low serum high-density lipoprotein cholesterol) and arterial hypertension. Moreover, a prothrombotic and chronic inflammatory state, often is associated with MetS and added subsequently to its definition [16, 17]. As late as the 1990s, World Health Organization (WHO) defined MetS as insulin resistance or diabetes or impaired glucose tolerance or impaired fasting glucose, plus two of these conditions: body mass index ≥30kg/m^2^ or waist/hip ratio >0,9 (man) / >0,85 (woman); blood pressure ≥140/90 mmHg or antihypertensive treatment; triglycerides ≥1,7 mmol/l (150 mg/dl) or lipid-lowering treatment; HDL<0,9 mmol/l (35 mg/dl) (man)<1,0 (39 mg/dl) (woman); albuminuria [18].

Later the National Cholesterol Education Program's Adult Treatment Panel III report (NCEP ATP III) and International Diabetes Federation (IDF) proposed a new set of criteria with ethnic/racial specific cut-offs to define MetS. This study group gave less attention to the criteria albuminuria and focused on abdominal obesity expressed as waist circumference. In particular, IDF considers waist circumference as essential criteria of MetS. The main difference between NCEP-ATP III and IDF MetS definition consists in the cut-offs chosen. The definitions of the MetS are summarized in Table 1. The concept of MetS has been disputed [19, 20], it is still recognized as a major risk factor cluster for CV disease, even in older subjects in whom MetS confers a 38% higher risk of myocardial infarction and stroke [21–23].

**Table 1:** Main definitions of Metabolic Syndrome

WH0 (1998) [15]	NCEP/ATP III (2001-2005) [257]	IDF (2005) [258]
Insulin resistance or diabetes or impaired glucose tolerance or impaired fasting glucose, plus two of these conditions	At least 3 of the following:	Abdominal obesity defined as waist circumference ≥ 94 cm in men and ≥80 cm in womenPlus at least two of the following criteria:
✓ Body mass index ≥30kg/m^2^ or waist ratio >0,9 man/ 0,85woman;	✓ Waist circumferences ≥ 102cm man and 88 woman;	✓ Increased fasting tryglicerides > 150 mg/dl (≥1,7 mmol/l) or lipid lowering treatment;
✓ Blood pressure≥ 140/90 mmHg or antihypertensive treatment;	✓ Blood pressure ≥ 130/85 mmHg, or antihypertensive treatment;	✓ HDL < 0,9 mmol/l (40 mg/dl) man <1,1 (50 mg/dl) women or lipid lowering treatment;
✓ Triglycerides≥ 1,7 mmol/(150 mg/dl) or lipid lowering treatment;	✓ Triglycerides ≥ 1,7 mmol/(150 mg/dl) or lipid lowering treatment;	✓ Increased blood pressure ≥ 130/85 mmHg, or antihypertensive treatment;
✓ HDL < 0,9 mmol/l (35 mg/dl) man <1,0 (39 mg/dl) woman;	✓ HDL < 1 mmol/l (39 mg/dl) man <1,3 (50 mg/dl) woman;	✓ High level of fasting glucose > 100 mg/dl (5,6 mmol/l), or T2DM;
✓ Glucose 5,6 mmol/dl (101 mg/dl) or hypoglycemic treatment;	✓ Glucose 5,6 mmol/dl (101 mg/dl) or hypoglycemic treatment;	
✓ Microalbuminuria;		
**Legend Table 1**: HDL: High Density Lipoprotein IDF: International Diabetes Federation NCEP ATP III: National Cholesterol Education Program's Adult Treatment Panel III report T2DM: type 2 diabetes mellitus WHO: World Healt Organization		

Main definitions drawn up by the WHO, NCEP/ ATP III and IDF of MetS recognized by the scientific world.

Despite the increased urinary albumin excretion (UAE) is not currently reported in diagnostic criteria of the MetS, recent studies had showed that albuminuria is considered an important indicator of cardiovascular risk. Actually, albuminuria and estimated glomerular filtration rate (eGFR) are considered not only biomarkers of progression and diagnosis of chronic Kidney disease (CKD) but also are significantly correlated with MetS and its components [24, 25]. In particular, a recent study proposed to reintroduce, in the diagnostic criteria of MetS, the albuminuria and elevated high-sensitive C- Reactive Protein (hs-CRP) [26]. In fact, albuminuria and hs-CRP are widely recognized as biomarkers of systemic endothelial dysfunction and low-grade chronic inflammation, respectively. According to guidelines, elevated UAE was defined as UAE ≥30 mg/24h and high hs-CRP as a hs-CRP≥3 mg/l [22, 23]. Elevated UAE is associated to higher risk of new-onset T2DM, CVD and CKD, whereas hs-CRP is only correlated to new onset CVD and CKD, but not with the onset of T2DM [27]. In fact albuminuria is not only a complication of T2DM but also can precede it [27–30]. In line with these studies, it has been suggested that since the birth, UAE levels are genetically determined and influenced by factors present in the intra-uterine environmental. These values represent both the state vascular and the individual susceptibility to organ damage [26–31].

### Phenotypes of obesity

Obesity can be categorised according to four phenotypes: 1) Normal weight obese (NOW) [32]; 2) Metabolically obese normal weight (MONW) [33, 34]; 3) Metabolically healthy obese (MHO); and 4) Metabolically unhealthy obese (MUO) or “at risk obese” with MetS [35]. Furthermore, the sarcopenic obesity was described and related to all these obesity phenotypes [36]. *NWO* syndrome was defined firstly by De Lorenzo et al, in order to indicate subjects with normal Body Mass Index (BMI) (18.5-24.9 kg/m^2^) and high body fat percentage (PBF%) (man ≥ 23.5%, woman ≥ 29.2%). They develop early oxidative stress and chronic inflammatory status and they have higher risk of cardio-metabolic diseases and sarcopenic obesity [37]. The inflammation is explained by the presence of single nucleotides polymorphisms (SNPs) in specific genes, such as -174G/C promoter polymorphism of the IL-6 gene that correlates to insulin resistance [38, 39], polymorphism in IL-15 receptor alpha, in methylenetetrahydrofolate reductase (MTHFR) genes [40] and in G/A -308 Tumor Necrosis Factor-alpha (TNF-α). The latter is associated to sarcopenic obesity [41]. *MONW* subjects have a normal weight but metabolic alterations typical of MetS (such as high blood pressure, low insulin sensitivity and dyslipidemia) [42, 43]. The higher amount of visceral fat in this phenotype leads to higher blood levels of inflammation cytokines (TNF-α and Interleukin-6 (IL-6) [44]. *MHO*, although have an excess of body fat, are metabolically healthy individuals. In fact, 20-30% of obese adults do not show the metabolic complication associated with obesity. MHO individuals have high insulin-sensitivity, normal blood pressure, no signs of chronic inflammation and normal lipid profile. Therefore, the inflammation is correlated to increasing of adipose tissue but is not always linked to obesity [45, 46]. *MUO*, are also called “at risk” obese subjects. They are obese for both anthropometric parameters (BMI ≥ 30 kg/m^2^) and fat mass percentage (PBF > 30%) and develop metabolic abnormalities such as MetS, T2DM and atherosclerotic CVD [47, 48]. This phenotype is linked to higher visceral adiposity than subcutaneous adiposity and consequently to a greater fatty acids release into the portal circulation and major deposition in liver [49].

The concept of *Sarcopenic Obesity* was introduced firstly by Roubenoff and co-workers [36], who suggested that the inflammation of visceral fat promotes muscle catabolism leading to a loss of muscle mass and muscle weakness [50]. Schrager et al demonstrated that sarcopenic obesity was directly related to higher levels of IL-6, CRP, IL-1 receptor antagonist and IL-7; consequently these cytokines were involved in both the development and progression of sarcopenic obesity [51]. The identification of obesity phenotypes can allow early detection the subjects at risk to develop the MetS in order to carry out timely personalized diet and drug treatment intervention.

### Adiposopathy and inflammation

The complexity of the “phenotype” MetS is related to two pathogenetic factors: adipose tissue (AT) and inflammation. AT is now considered not only a fat storage, but an active endocrine organ. It produces inflammatory cytokines, such as leptin, adiponectin, TNF-α, IL-1, IL-6 and pro-coagulant substances (PAI-1) that are associated with a chronic low–grade inflammation. Also the AT can produce vasoactive factors and molecules promoting insulin resistance (IR): free fatty acids (FFA), resistin and retinol binding protein 4 [52]. The AT consists of two compartments: brown adipose tissue (BAT), which is associated with thermogenesis, and white adipose tissue (WAT), lipid storage used during periods of nutrient deprivation. In literature, BAT was considered active only in infants and its thermogenic role finished after early months of life. However, recent findings have established that the BAT activity increases from childhood to adolescence with a peak at 13 years [53] and that BAT functional deposits are still present in adulthood [54]. Several studies have shown that exposure to cold in healthy volunteers rapidly increases the production of BAT, accumulating mainly in supraclavicular, laterocervical, paraspinal and mediastinal sites [55]. The authors have found also an endocrine function of BAT even if further analysis are needed to explain its ability to turn into WAT and its correlation with obesity [56]. The main role of WAT is energy and fat storage, however, it is also considered an endocrine organ. The WAT endocrine and deposit activity is largely determined in the early stages of life and is essential for its development and its pro-inflammatory property [57]. From the young age, the adipokines released by WAT are associated with the major cardio-metabolic complications of obesity (such as IR, elevated arterial blood pressure, dyslipidemia, T2DM and atherosclerosis).

During life, there are “permissive windows” in which the cells, tissues and organs are more sensitive to external signals. These periods particularly sensitive for the proliferation and differentiation of adiposities are the first year of life, years three to five (known as adiposity rebound period) and years nine to thirteen (puberty) [58, 59]. In turn WAT is divided in: visceral adipose tissue (VAT) and subcutaneous adipose tissue (SAT). The first is formed by mesenteric and omental fat and other deposits are localized in epicardial region and the latter is localized over the entire body surface, particularly in woman in breast and gluteo-femoral regions while in man in abdomen site. Although the VAT represents only 10-20% of the total fat, it is more associated with cardio-metabolic diseases [60]. Its pathogenic role is related to its location: VAT is more vascularized compared to SAT, releases a larger share of pro-inflammatory cytokines (as IL-6, IL-8, and TNF-α) and fatty acids in the blood stream. In fact, the “portal hypothesis” asserts that VAT is linked to an increased cardiovascular risk because fatty acids are poured in the portal blood and cause IR and vascular damage. Consequently, in according to Framingham Hearth Study, VAT is considered as an independent risk factor of CVD [61].

During positive caloric balance, adipocytes initially become hypertrophic, trigging paracrine adipogenic signaling in order to recruit other fat cells and to maintain AT physiologic functions during increased energy storage [62–64]. When the positive energy balance persists, adipogenesis is impaired causing adipocyte dysfunction [65]. The anatomic/functional alterations of AT, promoted by positive caloric balance, in genetically and environmentally susceptible individuals, are responsible of “adiposopathy” or “sick fat”. The endocrine and immune responses caused by the “adiposopathy” increase and/or exacerbate metabolic disorders and CVD risk factors (such as T2DM, high blood pressure and dyslipidemia) [66]. Furthermore, an increased fat storage induces adipocyte hypertrophy and intracellular hypoxia, causing the release of FFA in the blood. The “spill over” of lipids from dysfunctional adipocyte [67] lead to ectopic fat deposition in non adipose tissue/organ (such as liver, muscle, pancreas, kidney and blood vessels) causing “lipotoxicity”. In the muscle, lipotoxicity causes IR and in the pancreas reduces insulin secretion [66]. This process is the base of “meta-inflammation”, a chronic low-grade inflammatory state caused by obesity [68]. The lipid storage capacity decrease inhibits preadipocytes differentiation and increases lipolysis. In particular, TNF-α stimulates lipolysis in adipocyte by impairing the function of two enzymes, the hormone-sensitive lipase (HSL) and the fatty triglyceride lipase (ATGL). In this scenario, the macrophage infiltration in AT grows proportionally to BMI, fat mass and adipocyte hypertrophy and is a reversible process in obese patients who lose weight. The chemo-attractant factors such as monocyte-chemotactic protein-1 (MCP-1), chemerin, progranulin and colony stimulating factor-1 (CSF-1) allow the recruitment of macrophages from the bloodstream into AT [69] and the shift from a profile mainly anti-inflammatory (M2 macrophages) to a pro-inflammatory profile (M1 macrophages) [68]. M1 macrophages induce aerobic glycolysis and they are linked to inflammation and IR. M2 macrophages active oxidative metabolism and release anti-inflammatory cytokines [70].

The greater availability of circulating FFA produces storages outside the AT, causing cell dysfunction and cell death. This process (lipotoxicity) is involved in beta cell loss during the progression of T2DM (contributing to insulinopenia) and in the pathogenesis of complications related to T2DM injuring cardiomyocytes, hepatocytes, kidney parenchimal cells and endothelial cells [71, 72]. Recently, several deposits of adipose ectopic tissue are discovered in transgenic animal models [73]. One of the most involved organs in ectopic fat deposition is the liver, that is fundamental for oxidation and metabolism of FFA. During a positive caloric balance adipocyte hypertrophy and accumulation of VAT may contribute to enhance the flow of FFA to the liver, causing liver steatosis. Patients “inflexible” to hepatic metabolism of fatty acids are more prone to the accumulation of lipids in the liver and, consequently, the development of IR and dyslipidemia [64, 74]. Ectopic fats depots are also localized in the renal sinus (RS), where they may compress the renal vein and artery, increase kidney interstitial pressure and decrease sodium excretion. This condition is responsible for both arterial hypertension and CKD [75–77]. Several studies [78, 79] have recently demonstrated an ectopic adipose depot in epicardial region. The Epicardial Adipose Tissue (EAT) is metabolically active and located between the heart and pericardium. Anatomically it presents a prevalent localization around the right ventricle [80]. EAT produces several bioactive adipokines, as well as pro-inflammatory cytokines and pro-atherogenic substances such as TNF-α, IL-6, resistin, visfatin, omentin, leptin, PAI-1 and angiotensinogen. EAT is also associated with T2DM and obesity [81]. Under physiological conditions, the EAT plays a cardio-protective role through the local secretion of adipokines with anti-inflammatory and anti-atherogenic proprieties, as adiponectin and adrenomedullin. However in pathological conditions, the increase in thickness of the epicardial fat reflects the expansion of adipose tissue and visceral infarction, contributing to the development of MetS and coronary artery disease. Given its significant clinical implication, the evaluation of the EAT has been proposed as therapeutic target during the diet therapy or during pharmacological treatments directly or indirectly in order to weight loss [82]. During the redistribution of fat depots, adiposity is located undereath the deep fascia of the thigh and inside the muscles. This ectopic fat depot, called intermuscular adipose tissue (IMAT), is linked to insulin sensitivity in obese individual [83]. IMAT is also a significant predictor of both muscle and mobility function in older adults [84]. In this scenario, MetS cannot be related only to the increase in BMI but also to quantitative and qualitative enhancement of the AT and its localization.

### Nutrigenetics and nutrigenomic

Nutrition is probably the principal environmental factor that conditions the expression of genes involved in MetS. Nutrigenomics is defined as a prospective analysis of the differences between nutrients in relation to their impact on gene expression and finally on human health. Nutrigenomics allows us to study the interactions between nutrients and genes in order to establish a diet program according to the individual genetic profile and represents a tool to understand how the components of a particular diet (bioactive compounds) can alter gene expression, through their down and-or up regulation. The Nutrigenomics is a useful instrument in the prevention of Non-Transmissible Chronic Diseases (NTCDs) [85]. Nutrigenetics is a retrospective analysis of people genetic variations and their clinical response to specific nutrients. The term nutrigenetics was introduced by Brennan in 1975 [86]. Nutrigenetics approach was widely studied: the inter-individual differences in response to dietary factors underline the role of genes. Nutrient intake is manipulated or optimized according to an individuals’ genetic profile in order to reduce the risk to onset disease. The relationship between nutrigenetics and obesity, MetS and T2DM is largely based on data relating to dietary fat [87–89]. Sedentary lifestyle behaviour and a greater availability by high-calorie foods, in particular high fat food, are associated to obesity and CVD. The “thrifty genes” play a central role in the development of obesity and metabolic disorders. In the past, these genes promoted fat deposition as energy storage during times of food deprivation. Actually, in a modern environment of physical inactivity and excessive caloric consumption, the thrifty genes are associated to obesity and T2DM [90, 91]. Several studies support the interaction between genes and environment. The different nutritional responses are caused by SNPs mutations that occur in a single code base of a gene. They are very frequent: 1 SNP for 1,91 Kb DNA sequence and 5% of all cases of obesity and diabetes has been associated with monogenic disorders. The mutated genes, related to obesity, include leptin (LEP), leptin receptor (LEPR), pro-opiomelanocortin (POMC) and melanocortin-4 receptor (MC4R) [92]. Genome-wide association studies (GWAS) have evaluated susceptibility genes of T2DM and obesity. In particular, Calpain 10 (CAPN10), that encodes cysteine protease calpain 10, has been the first identified T2DM susceptibility gene [93]. Also, two Single Nucleotides polymorphism (SNPs) are associated with T2DM risk: transcription factor 7-like 2 (TCF7L2) and fat mass and obesity-associated (FTO) gene on chromosome 16. TCF7L2 polymorphisms is related to high risk of dyslipidemia and increased waist circumference. While the homozygous mutation of FTO gene (FTO rs9939609 mutation) is associated to 3 kg heavier and had 1,7 fold increased risk of obesity than the homozygous non-risk allele carriers [94]. Actually, FTO rs9939609 SNP is considered one of the most important gene variants predisposing to obesity [95] and the LIPGENE-SU.VI MAX study showed that FTO rs9939609 is also associated with overweight and abdominally obesity [96].

Numerous genes are also linked to an increased susceptibility to dyslipidemia, particularly peroxisome proliferator-activated receptor γ (PPARγ), a nuclear receptor that regulates adipocyte differentiation, lipid storage, fat-specific gene expression and insulin action. The most prevalent SNPs variants of PPAR*γ* gene identified is the Pro12Ala, correlated to T2DM, obesity, and other clinical disorders [97, 98]. Patients with T2DM carrying the Pro12Ala polymorphism have higher risk of obesity than non-carriers although the same energy intake, perhaps secondary to a better insulin sensitivity [99]. LIPGENE-SU.VI.MAX study shows that not only mutation in glucose metabolism but also in fatty acid metabolism cause IR and dyslipidemia, as evidenced by mutations in long-chain acyl CoA synthetase 1 (ACSL1) gene that is an important enzyme for mitochondrial beta-oxidation of long chain fatty acids. The polymorphisms (rs4862417, rs6552828, rs13120078, rs9997745 and rs12503643) in this locus genes increase MetS risk. Furthermore, a low fat dietary or a total dietary polynsatured fatty acids (PUFA) intake higher to 50^th^ percentile decrease the MetS risk in (G+) carriers [100]. The same study indicates that GG homozygotes of rs3790433 SNP at the LEPR gene have higher IR and increased MetS risk. The latter is exacerbated by a diet rich in n-6 PUFA and poor in n-3 PUFA, whereas a high n-3 or low n-6 PUFA background reduces the likelihood of developing MetS [101]. Both obesity and the MetS are characterized by a low-grade inflammatory state that causes or exacerbates their co-morbidities. A proper nutrigenomic approach is useful for reducing this inflammation. In fact, foods containing anti-inflammatory bioactives, such as caffeic acid (Yerba mate), tyrosol (olive oil), quercetin (fruits and vegetables), licopene (tomatoes an watermelon) and α-tocoferol (green tea) are able to reduce the inflammation. Its reduction is led by lower expression of ciclo-oxigenase-2 (COX-2) and inducible nitric oxide synthase (iNOS) genes after the inhibition of Kappa B-nuclear factor's translocation from the cytoplasm to the nucleus [102, 103]. Actually nutrigenomics, with the other “omics” sciences (such as Proteomics, Metabolomics and Transcriptomics), represents a pivotal tool in evaluating, treating and preventing different future diseases, especially in the area of NTCDs [85].

Nutrigenetic can be used to compose a personalized diet and in this scenario, an interesting study of Arkadianos et al. have shown that the inclusion of genetic information to personalize patient's diet improves the long term BMI reduction and blood glucose levels. In particular, the authors examined in 24 patients, the variants of 19 genes involved in the metabolism, such as methylenetetrahyidrofolate reductase (MTHFR), glutathione S-transferase (GST), superoxide-dismutase (SOD) 2 and 3 and vitamin D receptor (VDR) polymorphisms [104].

De Lorenzo et al., in other paper, have demonstrated that T (+) carriers in C677T MTHFR polymorphism have higher cardiovascular disease risk, osteoporosis and sarcopenia. In particular, the authors examined the impact of the C677T MTHFR gene polymorphism on body composition change induced by a balance hypocaloric Italian Mediterranean Diet. Before the nutritional intervention, T (+) carriers patients were fatter than T (-) carriers and after the nutritional intervention T (+) carriers had lower increase in total body lean/total body fat ratio compared to T (-) carriers [105]. The increased CV risk for T (+) carriers is due to hyperhomocysteinemia related in turn to lack of 5-methyltetrahydrofolate, necessary to methylate homocysteine (Hcy) into methionine.

Hyperhomocysteinemia is also associated to neural tube defects [106], Alzheimer's disease [107] and osteoporosis. The MTHFR polymorphism effects on bone mineral density (BMD) depend on folate status. The mechanism mediating this association, however, remains unclear, but may be partially caused by homocysteine effects on bone [108]. The nutrigenomic intervention by the supplementation of Folic acid and Vitamin B 12 is useful to reduce and, in some case, to normalize plasma Hcy concentrations. A meta-analysis of 25 randomized controlled trials, involving 2596 subjects, assessed that daily dose of ≥0.8 mg folic acid is able to induce the maximal reduction in plasma Hcy levels and the dose between 0.2 and 0.4 mg is associated with 60% and 90%, respectively, of this maximal effect [109]. Moreover the alternating vitamin treatment with folic acid and vitamin B12 confirmed the importance of folate therapy and secondary contribution of vitamin B12 in lowering Hcy also in haemodialysis patients with MTHFR genotype [110] (Table 2).

**Table 2:** Interaction genes and nutrients

Biological Process	Gene	Polymorphism	Genotype	Biomarker	Nutritional Factor	Dose genotype dependent
**INFLAMMATION**	MTHFR	677 C/T	C/C	Homocysteine	Folic Acid	200 (μg/day)
			C/T			400 (μg/day)
			T/T			800 (μg/day)
	MTHFR	1298 A/C	AA	Homocysteine	Folic Acid	200 (μg/day)
			AC			400 (μg/day)
			CC			800 (μg/day)
	IL-6	−174 G/C	G/G	C-Reactive ProteinLipoxygenaseFibrinogen	Ù 3	1,5 (g/day)
			G/C			1,5-3 (g/day)
			C/C			1,5-3 (g/day)
	TNF- α	−308 G/A	G/G		Ù 3	1,5 (g/day)
			G/A			1,5-3 (g/day)
			A/A			1,5-3 (g/day)
**STRESS OXIDATIVE**	SOD2	−28 C/T	CC	LDL ossidateNO_2_/NO_3_ANPCORAC	Ù 3	1,5 (g/day)
			CT			1,5-3 (g/day)
			TT			1,5-3 (g/day)
	CYP1A2	−163 A/C	AA		Ù 3	1,5 (g/day)
			AC			1,5-3 (g/day)
			CC			1,5-3 (g/day)
	GSTM1	ID	II		Ù 3	1,5 (g/day)
			ID			1,5-3 (g/day)
			DD			1,5-3 (g/day)
**LIPIDS METABOLISM**	APOA1	−75 G/A	GG	LDL oxidized	PUFA	< 4%
			GA			4 – 8%
			AA			> 8%
**Legend Table 2**: APOA1: Apolipoprotein A1 CYP1A2:Cytochrome P450 1A2 GSTM1: Glutathione S-transferase Mu 1 IL-6: interleukin-6 MTHFR: methylenetetrahydrofolate reductase SOD2: Superoxide dismutase 2 TNF-α: tumor necrosis factor alpha						

Personalized diet therapy intervention and nutrients dose change according to genetic polymorphisms.

### Impact of mediterranean diet on metabolic syndrome and obesity

The Seven Country Study, an International Cooperative Study on coronary heart disease epidemiology, promoted by the American physiologist Ancel Keys and the Italian Flaminio Fidanza, investigated the benefits of the MD on human health, comparing European rural cohorts (the Mediterranean cohorts Crete, Corfù, Crevalcore, Montegiorgio and Dalmatia) and the non–Mediterranean cohorts (East and West Finland, Slavonia and Velika Krsna).

**Figure 1:**
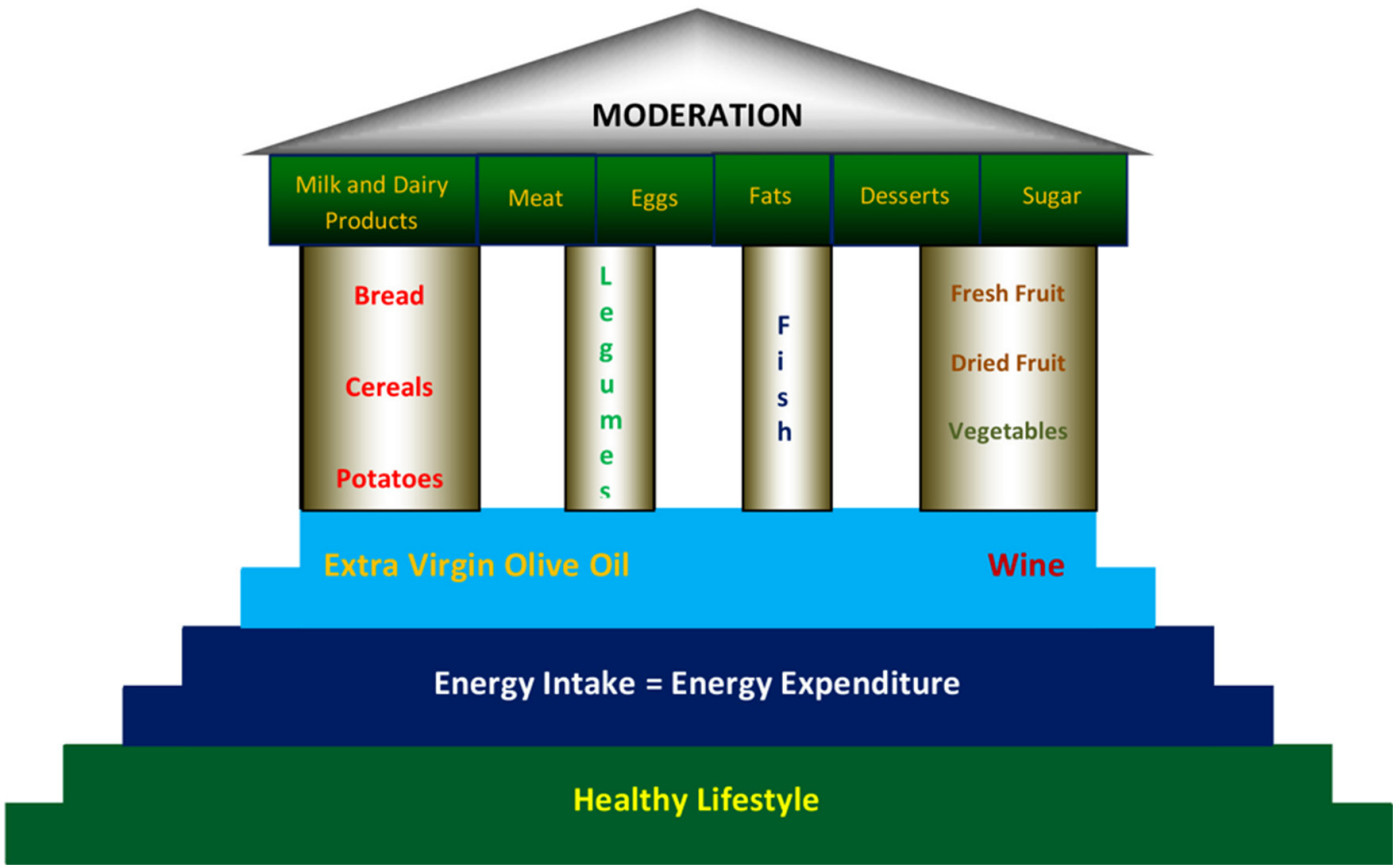
Representation of Mediterranean Diet by Paestum Temple Modified By De Lorenzo A, Fidanza F [89] As shown in the figure, at the base of the Mediterranean style there is a healthy lifestyle, energy intake equal to the expenditure, extra virgin olive oil and wine. Within the eating habits must be more present: cereals, legumes, fish, fresh fruits, dried fruits and vegetables. While animal foods and simple sugars should be limited use. Moderation should be the focal point of the Mediterranean model.

In order to assess the adhesion to the Healthy Reference National Mediterranean Diet (HRNMD), it was introduced the Mediterranean Adequacy Index (MAI). It is calculated by dividing the sum of total energy percentages of the food groups typical of HRNMD (bread, cereals, legumes, potatoes, vegetables, fresh fruit, nuts, fish, wine, vegetable oil), by the sum of the dietary energy percentage of food groups that are not characteristic of a HRNMD (milk, cheese, meat, eggs, animal fats and margarines, sweet beverages, cakes, pies, cookies and sugar). The membership of food to one of two groups is based on the results of a dietary survey carried out in 1960 in a sub-sample of Nicotera man aged 45-54 years. Nicotera was chosen for low frequency of cardiovascular diseases, T2DM and arterial hypertension [111–113]. The MAI was inversely associated with the 25-year death rates from cardiac heart disease (CHD). In particular, a 2.7point increase of MAI was associated with a CHD mortality decrease of 26% in 20 years of follow-ups and of 21% in 40 years of follow-ups in two Italian rural cohorts of the Seven Countries Study, Crevalcore and Montegiorgio. It was demonstrated that this association is independent from possible confounding factors, such as age, cigarette consumption, systolic blood pressure, serum cholesterol, physical activity and BMI [113]. A longitudinal study of Alberti-Fidanza et al. has evaluated how much near or far eating habits of population are from a reference MD. They were investigated men (aged 45-65 years) from rural areas of Italy in the Seven Countries Study for 26 years (Crevalcore and Montegiorgio), elderly men and women from Perugia for 11 years, men and women from Pollica (Salerno) for 32 years and families from Rofrano (Salerno) for 41 years. The median value of MAI among Nicotera's people was 7.2. The examination during four decades has showed that the diet over the years has abandoned the nutritional characteristics of the reference Italian-Mediterranean diet. In particular, in Nicotera, the median MAI of the diet examined in 1996 has decreased to 2.8, while in Crevalcore it was reduced from 2.9 in 1965 to 2.2 in 1991 and in Montegiorgio from 5.6 in 1965 to 3.9 in 1991. In elderly men from Perugia the median value of MAI was decreased from 4.9 in 1976 to 3.2 in 1987, while in women from 3.1 to 2.6 [114]. However, many others nutritional indices are proposed to evaluate the efficacy of MD on healthy status. Cholesterol/Saturated Fat Index (CSI) is associated to atherogenicity grade, whereas the Atherogenic Index (AI) and Thrombogenic Index (TI) have been proposed in order to evaluate the possible link between diet and CHD [115]. The Mediterranean Diet Score (MDS) was proposed by Trichopoulou et al. in 1995 for the purpose of examine the relationship between MD and CHD in a random sample of 1159 Jewish people in Israel. The study showed that higher MDS correlated to lower risk of myocardial infarction, coronary by-pass, angioplasty and any other cardiovascular disease [116]. In 2003 the MDS was modified by Trichopoulou et al., adding moderate fish consumption among the inclusion criteria. A two-points increase in the MDS with fish (t-MED) was associated with a reduction in CHD mortality by 33% [117–119] (Figure 1).

A US multiethnic population study has compared 4 diet-quality indexes, the Healthy Eating Index-2010 (HEI-2010), the Alternative HEI-2010 (AHEI-2010), the alternate Mediterranean Diet Score (aMED) and the Dietary Approaches to Stop Hypertension (DASH), in order to evaluate their effectiveness to predict the decrease risk of mortality for all causes. The authors suggested that high scores were inversely related to risk of mortality for CVD and cancer, reinforcing the concept that healthful diet is important to improve the quality of life and to provide a greater longevity [120].

In the 2000s, a Spanish multi-center, randomized trial of people at high risk for CVD, called PREDIMED (PREvenciòn con DIeta MEDiterrànea) study, showed that a MD supplemented with nuts reversed MetS more than a low-fat diet [121]. The study, conducted between October 2003 and December 2010, involved 7447 people divided into three subgroups: participants with MD supplemented with extra-virgin oil, participants with a MD supplemented with nuts and participants with low-fat diet (control group) [122]. The MD with extra-virgin oil after a median follow-up of 4.8 years, had reduced by 30% the rate of CVD events, whereas the MD with nuts had reduced by 28%, compared with the control group. Moreover the MD, either supplemented with extra virgin olive oil or nuts, was not associated with the onset of MetS but only with its regression [123].

A recent meta-analysis conducted by Grosso et al. has linked the association between MD adherence and CVD incidence and mortality. They found an important decrease of incidence of CHD, myocardial infarction and stroke. The most protective effects are associated to higher consumption of olive oil, fruits, vegetables, legumes, nuts and moderate intake of wine (especially red wine) [124].

The “ATTICA study” assessed the effect of the MD on total antioxidant capacity in 3042 healthy subjects, selected from the Attica area of Greece. The adherence to MD was evaluated with a diet score. Total antioxidant capacity (TAC) was measured with colorimetric test on serum samples taken from the participants. The study has demonstrated that greater adherence to the MD is associated to higher TAC levels and low oxidized LDL-cholesterol concentrations, suggesting a healthy role of MD on the cardiovascular system [125].

It's known that the adherence to MD and the change of unhealthy lifestyle have a great impact on the individual components of the MetS. In fact, the MD can be considered the first step in the treatment of NTCDs [126–128].

De Lorenzo et al had studied the impact of a moderately hypocaloric Mediterranean Diet (MHMD) on body composition and metabolic profile in 19 obese women. After the 2-months MHMD regimen, total fat mass and the segmental fat mass from trunk and legs were significantly decreased, while no significant loss of total and segmental lean body mass was observed. In the metabolic profile, a significant decrease of basal insulin, total and LDL-cholesterol, uric acid and fibrinogen was observed, while any change in fasting blood glucose, HDL-cholesterol and triglycerides was reported. This study has demonstrated that a MHMD with adequate nutritional indices based on foods and food combinations, was associated to a high effectiveness in term of compliance and safety, in particular to prevention of loss of fat-free mass and on metabolic parameters in obese women [115]. Andreoli et al. verified the effect of MHMD and physical activity on the body cell mass (BCM) and cardiovascular risk factors in obese women. After 2 and 4 months, the BCM has remained stable while the body composition has improved and major cardiovascular risk factors decreased [129, 130]. A recent paper had examined the effect of Italian Mediterranean Diet (IMD) and Italian Mediterranean Organic Diet (IMOD), in healthy subjects and in CKD patients, on body composition and on laboratory parameters. The aims of this study were to evaluate the impact of IMD and IMOD on decrease of cardiovascular risk factors and on the progression of renal disease. The authors demonstrated that the IMOD diet, according to “Nicotera Diet”, was able to reduce the total Hcy, phosphorus and albuminuria levels in CKD patients and to ameliorate the cardiovascular risk profile in both population examined [131]. Another study of the same authors highlighted that IMD and IMOD diets represent a valid nutritional alternative intervention to low-protein diet in CKD patients on conservative therapy [132], since the low protein diet would seem to be a contributory cause of sarcopenia in these patients [133]. An interesting study of Di Daniele et al has shown that the IMD promotes weight loss and reduces the growing burden of cardiovascular risk factors that typifies patients with MetS. Specifically, a balanced hypocaloric IMD improves significantly systolic and diastolic blood pressure and fasting glucose in 80 white Italian subjects with MetS. Moreover the authors have observed a resolution of MetS in 52% of treated patients [134].

The apparent capacity of the traditional MD to reduce the risk of development and progression of CVD, cancer and degenerative diseases has been attributed, at least in part, to nutraceutical effect of micronutrients and compounds with capacity antithrombotic, anticancer and antioxidant. Carbohydrates are represented predominantly by starch, provided in large part from wheat (bread, pasta) and in smaller quantities from other cereals and pulses, while the proportion of sucrose, for the moderate consumption of sugar and sweets as such, is very low. Fish products and the extra virgin olive oil are mainly responsible for the contribution of fatty acids essential and oleic acid. The share of energy from oleic acid (monounsaturated) in the context of fatty acids is the largest one in the MD, which can also exceed 15% of the energy. Energy from saturated fatty acids (SFA) is not more than 7% of the total energy, so that the ratio polyunsaturated fatty acids / saturated is about 1:1. In the context of fatty acids essential, ω3/ω6 ratio is very important. Fatty acids ω3 play an important role in the prevention and treatment not only of CVD but also cancer, rheumatoid arthritis, psoriasis and cataract. The daily ω3 recommended dose is 1,5 g in adult man and 1 g in adult woman [135, 136] (Table 3).

**Table 3:** Nutritional factors and targets

Nutritional factors	Targets
Total Fat	15-30%
Saturated fatty acids	<10%
Polinsatured fatty acids (PUFA)	6-10%
Polinsatured fatty acids n 3 (PUFA)	5-8%
Polinsatured fatty acids n 6 (PUFA)	1-2%
**Trans fatty acids**	<1%
Monoinsatured fatty acids (MUFA)	*
Total Carbohydrates	55-75%
Carbohydrates simple	<10%
Proteins	10-15%
Cholesterol	<300 mg/day
Sodium Chloride	<5 g/day (<2 g/day)
Vegetables and Fruits	≥ 400 g/day
Flavonois	> 50 mg/kg
Total dietary fiber	> 25-30 g/day
Non-starch polysaccharides	> 20 g/day
Mediterranean Adequacy Index (MAI)	>6,5
**Legend Table 3**: PUFA: Polinsatured fatty acids MUFA: Monoinsatured fatty acids MAI: Mediterranean Adequacy Index	

Ranges of values for the nutritional targets in the general population according to Mediterranean Diet.

* This value is calculated as total fat - (satured fatty acids + polynsatured fatty acids + trans fatty acids)

In LIPGENE study, a large pan-European isocaloric dietary intervention study of MetS subjects, SFA have been replaced with MUFA or low-fat, high amount of complex carbohydrate, proving an effective improvement of insulin sensitivity only in patients whose habitual, pre-intervention dietary, fat intake was below the median (<36% energy from fat) [137]. Another study, called MUFA in Obesity (MUFObes), shows that MUFA rich diet improves insulin and glucose concentrations and reduces the risk of weight regain [138, 139]. In the KANWU (Kopio, Aahhus, Naples, Wollongon and Uppsala) Study, a change in the proportions of dietary fatty acid, decreasing SFA and increasing MUFA, leads to an improvement of insulin sensitivity [140].

The Mediterranean Diet is rich of ω-3 PUFA, contained primarily in fish and seafood. Eicosapentaenoic acids (EPA, C20:5, n-3) and docosahexaenoic acid (DHA, C22:6, n-3) are the most important PUFA associated to cardio-protective effects [141].

Several intervention studies such as the “Gruppo Italiano per lo Studio della Sopravvivenza nell’Infarto Miocardico” (GISSI- Prevenzione trial and the Cardiovascular Health Study) have demonstrated that enhanced intake of EPA and DHA improves the risk of CHD [130–132]. The cardio-protective effects of EPA and DHA can be explained by their modulate K, Na, and Ca channels activities in myocardial cells, regulating myocyte electrical excitability and contractility. These effects are concentration-dependent [142, 143]. The role of n-6 PUFAs on human health is not clearly identified. However, both n-3 and n-6 PUFAs seem to be positive effect on cardiovascular disease, cancer, and depression in humans [144]. Many of the characteristic components of the traditional MD are known to have positive effects on the health status and they are called “functional foods”. Carotenoids, folic acid and fibers would appear an important role in the prevention of CHD. Consumption vegetables, an important source of phytosterols, is associated with a reduction in the level of cholesterol in serum and cardiovascular risk. The increased consumption of fruits and vegetables, containing phytoestrogenic substances, may offer an alternative to hormonal therapy in menopausal women. In the gut, these residues are converted into estrogens, neutralizing the typical menopausal hormone deprivation. The polyphenols in *red wine* have antioxidant activity and cytoprotective action and induce a change in the lipoprotein profile, in platelet aggregation, and in redox mechanisms. The wine and other derivatives of red grapes rich in resveratrol (a polyphenol stilbene) determine a vasodilatory effect through the endothelium-dependent up-regulation of nitric oxide (NO) production, and have a significant antioxidant activity. Resveratrol, found in grape skins, is a potent anti-inflammatory by inhibiting iNOS, COX-2 and NF-kB. It was suggested that the histone acetylation, activated by NF-kB, could be suppressed by resveratrol [145]. The antioxidant capacity of red wine is higher than antioxidant capacity of white wine because is an excellent source of polyphenolic compounds such as phenolic acids, flavonoids [146]. Di Renzo et al have shown that the red wine combined with different meals (such as McDonald's Meal and a Mediterranean Meal) had a positive effect on ox-LDL and antioxidant gene expression (catalase, superoxide dismutase 2, sirtuins 2 and glutathione peroxidase 1). Thanks to its antioxidant capacity the red wine combined with MD may be an essential component of a holistic approach to prevent NTCDs linked to inflammation [147]. Several studies confirmed that flavonoids, especially resveratrol, inhibit pre-adipocyte proliferation, adipogenic differentiation and *de novo* lipogenesis [134, 148, 149]. In the PREDIMED population the moderate red wine consumption (≥ 1 drink/d) is associated with a lower prevalence of the MetS in patients at a high cardiovascular risk [150].

Another typical food of MD, besides the red wine, with antioxidant capacity is the *tomato*. Because the lycopene, a carotene phytochemical containing in tomato, induces the up-regulation of anti-oxidant enzymes activity (SOD, GPX and catalase) and shows anti-inflammatory properties and insulin-sensitizing [151]. Thanks its characteristics mitigates inflammation related to obesity. Ghavipour et al. have highlighted that daily supplementation with one glass of tomato juice decreases inflammatory cytokines such as TNF-α, IL-6 and IL-8 after 20 days of assumption [152]. This result was confirmed by an another study of McEneny et al, in overweight subjects, in which a lycopene supplementation for 12 weeks decreased systemic levels of serum amyloid A [153]. Moreover, the daily tomato juice supplementation ameliorates also the body composition: a study on 30 young females with BMI≥20 kg/m^2^ demonstrated that 280 ml/day of tomato juice for two months significantly reduce body weight, body fat waist circumferences, BMI and serum levels of cholesterol, monocyte chemoattractant protein-1 (MCP-1) while significantly increased serum levels of adiponectin, triglycerides and lycopene [154]. The tomato and its products contain some natural compounds (such as chlorogenic, caffeic, ferulic and p-coumaric acids) that may inhibit platelet activation. These anti-platelet and anti-thrombotic activities of tomato were not modified by industrial tomato processing [155].

Another potential food promoting the positive effect of the MD is the *extra-virgin olive oil*, in fact its bioactive components would seem to have endothelium-protective and anti-oxidative properties [156]. A systematic review suggested that markers of inflammation such as C-reactive protein, IL-6 and those related to endothelial-function such as flow mediated dilatation and E-selectine, are significantly ameliorated after supplementation of extra-virgin olive oil [157]. The polyphenol intake is associated with lower cardiovascular mortality rates [158]. Extra-virgin olive polyphenols have strong antioxidant proprieties as demonstrated in experimental studies [159]. In healthy subjects and in cardiovascular and dyslipidemic patients, the polyphenols contained in extra-virgin olive oil improved ischemic reactive hyperemia blood pressure and inflammatory status [160]. The presence of extra-virgin olive oil in addition to raw vegetables increases the amount of phenolic components as oleuropein, pinoresinol, hydroxytyrosol and tyrosol and improves the contents of vegetable phenolics such as chlorogenic acid and rutin [161]. Moreover, olive oil is an excellent MUFA source and improves insulin sensitivity [162]. Estruch et al. compared the effects of MD, supplemented with either 1l g/week of virgin olive oil or 30 g/day of nuts and of low-fat diet on CVD markers. After 3 months of diet treatment, in patients with Mediterranean diet plus virgin olive oil or nuts was observed a reduction of ox-LDL levels, whereas any change occurred in low-fat diet group [163]. Moreover, MD reduces IR, thanks to the high fibers content [164]. *In vivo* recent study has shown that a diet rich in soluble fibers (20 g/1000 kcal) and a decreased consumption of food items with a high glycemic index decreases the prevalence of MetS by improving blood pressure (BP) and IR [165, 166].

Recently the focus has shifted on *caffeinated beverages*, such as coffee and tea, and their association with the components of MetS. A study conducted on 1889 inhabitants living in Sicily has demonstrated that the intake of coffee and tea reduced the prevalence of MetS and improved the components of MetS, lowering triglycerides and fasting plasma glucose. The antioxidant properties of coffee and tea are associated to the content of polyphenols; in particular, the main polyphenols are the isomers of chlorogenic acid contained in coffee and the catechin chemical family contained in tea [167].

The high consumption of *fresh fruits*, in particular blueberries, is related to anti-inflammatory property. In the human and animal studies, polyphenolic anthocyanins (containing in blueberries) ameliorated systolic BP because of significantly increase of endothelial NO synthase levels and decrease of vasoconstriction, via nitric oxide-mediate pathway, and the reduction of renal oxidative stress [168, 169].

Basu et al. showed that the daily blueberries supplementation (about 350 g fresh blueberries or 50 g freeze-dried blueberries) decrease significantly plasma ox-LDL, malondialdehyde (MDA) and hydroxynonenal concentrations in subjects with MetS after eight weeks of supplementation compared to control group. Moreover, during the study a significantly reduction of systolic and diastolic BP was observed [170]. This result was confirmed by another study that has demonstrated that regular bilberry consumption may reduce low-grade inflammation (decrease serum high sensitivity CRP, IL-6, IL-12 and lipopolysaccharides) characteristic of MetS [171]. Also strawberries have high antioxidant capacity due to polyphenolic anthocyanins, fibers and several micronutrients content. A supplementation for 8 weeks of 4 cups freeze-dried strawberry beverage in patients with MetS, improved atherosclerosis risk factors, in particular decrease total and LDL-cholesterol and levels of vascular cell adhesion-1 (VCAM1) [172]. Flavonoid-rich foods intake is inversely associated with risk of death for CVD, CHD and all causes in post-menopausal US women, after 16 years of follow-up. In this study the authors considered both total flavonoids intake and seven subclasses of flavonols (anthocyanidin, flavanones, flavones, flavonols, isoflavones, flavan-3-ols or monomers, proanthocyanidins). In the analyses of the food, apples and pears and red-wine are linked to a lower risk of CHD and CVD. Grapefruit, major source of flavonones, is also associated with a decrease risk of CHD mortality [173].

The healthy properties of the MD cannot be limited to any single nutrient, food or food component, but they have to be extended to the entire meal pattern and life-style (Table 4).

**Table 4:** List of studies about Mediterranean Diet

*ReferenceCountryYears*	*Population*	*Follow-up*	*NAge range*	*Outcome*	*FINDINGS*
Sala-Salvadò J, et al.-------**Spain**2003-2010	Community-dwelling men and women with no previously documented cardiovascular diseases	4.8 years	7447-------55-80	PREDIMED randomized trial analyzed the effect of Mediterranean Diet on incidence and reversion of metabolic syndrome.	The Mediterranean Diet with extra-virgin oil after a median follow-up of 4.8 years, had reduced by 30% the rate of CVD events. The Mediterranean Diet with nuts had reduced by 28%. The Mediterranean diet, either supplemented with extra virgin olive oil or nuts, was not associated with the onset of metabolic syndrome but only with its regression.
De Lorenzo A, et al.-------**Italy**2001	Obese Italian women without obesity-related complications or other diseases	2 months	19-------32±4	The study evaluated the efficacy and the safety of a moderately hypocaloric Mediterranean Diet (MHMD) on body composition and metabolic profile.	Total fat mass and segmental fat mass from trunk and legs were significantly decreased, while no significant loss of total and segmental lean body mass was observed. In the metabolic profile, a significant decrease of basal insulin, total and LDL-cholesterol, uric acid and fibrinogen was observed, while any change in fasting blood glucose, HDL-cholesterol and triglycerides was reported.
Andreoli A, et al.-------**Italy**2008	Obese women	4 months	47-------39.7±13	The study evaluated the effects of a moderately hypoenergetic Mediterranean diet (MHMD) and exercise program on body cell mass (BCM) and cardiovascular disease risk factors in obese women.	MHMD and exercise program for 4 month preserved BCM and improved cardiovascular disease risk factors in obese women
De Lorenzo A., et al.-------**Italy**2010	Caucasian Italian men (100 healthy male individuals and 50 male CKD patients)	14 days	150-------30-65	The aim was to verify the effect of Italian Mediterranean Diet (IMD) on body composition and biochemical parameters in healthy individuals and in Chronic Kidney Disease (CKD) patients, in order to decrease cardiovascular disease (CVD) risk factor and the progression of renal diseases.	The IMOD diet, according to “Nicotera Diet”, was able to reduce the total homocysteine, phosphorus and albuminuria levels in CKD patients and to ameliorate the cardiovascular risk profile in both population examined
Di Daniele N, et al.-------**Italy**2014	Caucasian Italian men with CKD and stable renal function	28 days	40-------42-54	The aim was to explore the effect of an Italian Mediterranean organic diet (IMOD) versus low-protein diet (LPD) in chronic kidney diseases (CKD) patients, according to patients’ carrier status for the methylenetetrahydrofolate reductase (MTHFR) C677T polymorphism.	IMD and IMOD diets represent a valid nutritional alternative intervention to low-protein diet in chronic kidney disease (CKD) patients on conservative therapy.
Di Renzo L, et al.-------**Italy**2014	Healthy volunteers aged 18-65 years and BMI ≥ 19 kg/m^2^	18 weeks	24-------18-65	The study evaluated if the consumption of a Mc Donald's Meal (McD) and a Mediterranean Meal (MM) with and without the addiction of red wine, reduces oxidized (ox-) LDL and the expression of oxidative and inflammatory genes.	When red wine is associated with McD or MM, values of ox-LDL are lowered, the expression of antioxidant genes is increased, while CCL5 expression is decreased.
Ghavipour M, et al.-------**Iran**2012	Overweight or obese female students	20 days	106-------20-40	The study verifies if the consumption of a lycopene-rich food can reduce inflammation in overweight or obese people.	Tomato juice reduces inflammation in overweight and obese females. Serum concentrations of IL-8 and TNF-α decreased significantly in overweight subjects. Among obese subjects, serum IL-6 concentration was decreased in the intervention group compared with the control group, with no differences in IL-8 and TNF-α observed.
Mc Eneny J, et al.-------**UK**2012	Moderately overweight individuals.	12 weeks	54-------middle-aged	This study examined lycopene's ability to lower systemic and high-density lipoprotein (HDL)-associated inflammation in moderately overweight middle-aged subjects.	A lycopene supplementation for 12 weeks decreased systemic levels of serum amyloid A.
Li YF, et al.-------**Taiwan**2015	Young females with BMI ≥ 30 kg/m^2^	2 months	25-------20-30	This study showed the effect of tomato juice's supplementation on indices linked to metabolic health and adipokine profiles in generally healthy people.	Daily tomato juice supplementation reduces waist circumference, as well as serum cholesterol and inflammatory adipokine levels in young healthy women and these results are unrelated to body fat changes.
Pitsavos C, et al.-------**Greece**2005	Random sample with no clinical evidence of cardiovascular disease	1 year	3042-------18-89	“the ATTICA study” assessed the effect of the MD on total antioxidant capacity (TAC)	Greater adherence to the MD is associated to higher TAC levels and low oxidized LDL-cholesterol concentrations.
Tierney, A.C.; et al.-------**EU**2011	MetS subjects	12 weeks	417-------Mean 54.9	In LIPGENE study, a large pan-European isocaloric dietary intervention study of MetS subjects, saturated fatty acid (SFA) have been replaced with MUFA or low-fat, high complex carbohydrate.	Improvement of insulin sensitivity only in patients whose habitual pre-intervention dietary fat intake was below the median (<36% energy from fat).
Vessby B, et al. & KANWU Study-------**Sweden**2001	Healthy subjects	3 months	162-------30-65	The aim was to evaluate whether a change in dietary fat quality could improve insulin action.	A decrease of saturated fatty acid and an increase of monounsaturated fatty acid, improves insulin sensitivity but has no effect on insulin secretion.
GISSI-Prevenzione trial.-------**Italy**1999	Patients surviving recent (≤3 months) myocardial infarction	3-5 years	11324-------50-80	The study investigated the effects of foods rich in vitamin E (α-tocopherol) and n-3 polyunsaturated fatty acids (PUFA) in patients who had myocardial infarction.	Dietary supplementation with n-3 PUFA led to a clinically important and satistically significant benefit. Vitamin E had no benefit.
Di Daniele N, et al.-------**Italy**2013	White Italian subjects with MS	6 months	80-------48,7± 13	The aim was to evaluate the benefits of dietary intervention based on a typical IMD on body composition, cardiometabolic changes and reduction in cardiovascular disease in patients with MS	The MS was resolved in 52% of the patients. Significant improvements in systolic and diastolic blood pressure and fasting glucose occurred.
Fitò M, et al.-------**Spain**2003-2004	Subjects with high cardiovascular risk	3 months	372-------55-80	The aim was to verify the efficacy of the Mediterranean diet (MD) on the primary prevention of coronary heart disease in patients with high cardiovascular risk.	After 3 months of Mediterranean Diet, individuals at high cardiovascular showed significant reductions in cellular lipid levels and LDL oxidation.
Cuenca-García M, et al.-------**Spain**1987-1999	Middle-aged healthy men and women	12 years	12449-------20-84	The study examined the association between three predefined dietary indices (Ideal Diet Index, Diet Quality Index, and Mediterranean Diet Score) and both cardiovascular disease (CVD) risk factors and long-term mortality in adult Aerobics Center Longitudinal Study's participants.	Higher Ideal Diet Index, Diet Quality Index, and Mediterranean Diet Score scores were significantly linked to lower body mass index, cholesterol and glucose levels, and diastolic blood pressure, and higher cardiorespiratory fitness.
Ruano J, et al.-------**Spain**2005	Hypercholesterolemic volunteers	2 hours	21-------53-68	The aim of this study was to evaluate the effects of the phenolic content of virgin olive oil on endothelial reactivity.	The intake of the polyphenol-rich breakfast was associated with a greater increase of nitrates/nitritis ratio and lower lipoperoxides and 8-epi prostaglandin-F2alpha levels.
Grosso G, et al.-------**Italy**2014	Subjects with or without metabolic syndrome	1 year	1889-------Mean 50.2	The study investigated the relationship between the beverages containing caffeine and the components of metabolic syndrome.	Coffee and tea consumption was significantly associated with reduced odds of MS; however, no direct association between caffeine intake and MS components was evaluated.
Basu A, et al.-------**US**2010	Men and women with metabolic syndrome	8 weeks	48-------50± 3	The study aimed to evaluate the effects of blueberry supplementation on features of metabolic syndrome, lipid peroxidation, and inflammation in obese men and women.	The blueberry supplementation decreases systolic and diastolic blood pressures, whereas the serum glucose concentration and lipid profiles were not affected.
Kolehmainen M et al.-------**Finland**2012	Subjects with metabolic syndrome	8 weeks	27-------53± 6	The aim was to study the impact of bilberries on inflammation and gene expression profile in peripheral blood mononuclear cells.	Blueberry supplementation decreases serum high-sensitivity C-reactive protein, IL-6, IL-12, and LPS concentration, demonstrating a decrease of cardiometabolic risk in long term.
Basu A et al.-------**US**2010	Subjects with metabolic syndrome	8 weeks	27-------47 ±3	The aim was to verify if a freeze-dried strawberry supplementation can improve blood pressure, impaired glucose, dyslipidemia, or circulating adhesion molecules in obese subjects with metabolic syndrome.	A short-term supplementation with 4 cups of freeze-dried strawberry beverage improve selected atherosclerotic risk factors, including dyslipidemia and circulating adhesion molecules in subjects with metabolic syndrome.
**Legend Table 4**: CVD: cardiovascular diseases MHMD: moderately hypocaloric Mediterranean Diet LDL: Low density lipopretein HDL: high density liporptein BCM: Body cellular mass IMD: Italian Mediterranean Diet IMOD: Italian Mediterranean Organic Diet CKD: Chronic kidney disease LPD: Low protein diet MTHFR: methylenetetrahydrofolate reductase McD: Mc Donald's Meal MM: Medierranean Meal CCL5:Chemokine (C-C motif) ligand 5 IL-8: interleukin-8 TNF-α: tumor necrosis factor alpha IL-6: interleukin-6 MD: Mediterranean Diet TAC: Total antioxidant capacity MetS: Metabolic Syndrome SFA: Saturated Fatty Acid MUFA: Monoinsaturated Fatty Acids MS: Metabolic Syndrome IL-12: interleukin-12 LPS: Lipopolysaccharide					

The most important studies in the world about the impact of Mediterranean Diet on cardiovascular risk factors: findings suggest the protective role of MD.

## MEDITERRANEAN DIET AND CANCER

The cancer, actually, is considered the second cause of death in the world after cardiovascular diseases [174].

Obesity is related to a higher risk of cancer because of chronic low-grade inflammation, induced by pro-inflammatory cytokines released by adipose tissue. Data indicated that inflammation is linked to oxidative stress. In fact Reactive Oxygen Species (ROS) are continuously produced by cellular and oxidative metabolism, and accumulation of ROS may cause oxidative damage and promote inflammatory reactions [175]. During carcinogenesis, immune and inflammation response produces cytokines and chemokines that facilitate cancer development, angiogenesis and modify tumor microenvironment [176]. These cytokines and chemokines can change expression of important transcription factors, including NF-kB, STAT-3, beta-catenin, p53, HIF-1 NFAT, responsible for cell response [177]. Among there, NF-kB is considered the crucial regulator in tumorigenesis, linking ageing, obesity, inflammation, and cancer [178].

Cancer cell shows a clear altered metabolism, with distinct usage of the energy versus the biosynthetic pathways [179–184]. A large number of studies have evaluated the ROS in cancer cells [185–196] and their relationship with the mitochondrial activity [197–208].

This energy and anabolic change implies also a significant interplay with autophagy [209–212], a pathway that strongly affects cancer progression, as well as distinct signalling pathways, such as for example JNK [212, 213], the p53 family [214–221] death receptors, as well as mechanisms [222, 223].

The diet and the natural antioxidants, in particular, play an important natural antioxidants play an important role on these metabolic pathways affecting cancer progression [224–228]. In keeping, several dietary supplements can offer ancillary support in cancer treatment [229–235].

The MD has a preventive action on cancer, because of the anti-proliferative and anti-apoptotic effects on cancer cell [236].

Noto et al. have demonstrated that, in a Mediterranean population, metabolic disturbances (obesity and MetS) are predictive of cancer in a 25 years follow up [237]. Epidemiological studies assessed that 640 million adults in 2014 and 110 million children and adolescents in 2013 were obese. The obesity-related cancer represents up to 9% of cancer among women in North America, Europe and the Middle East. In 2013, 4.5 million deaths worldwide were related to overweight and obesity [238]. The International Agency for Research on Cancer (IARC) working group concluded that excess of body fatness increases cancer risk, especially for colon, rectum, gastric cardia, liver, gallbladder, pancreas, kidney cancers and esophageal adenocarcinoma, as later confirmed by Beavis et al. in 2016 [239, 240].

In particular, cancer's risk increased from 1.2 to 1.5 in overweight individuals and from 1.5 to 1.8 in obesity people, especially for colon, gastric cardia, liver, gallbladder, pancreas and kidney cancers, while the relative risk for esophageal adenocarcinoma was up to 4.8 in patients with BMI ≥ 40 kg/m^2^ [241–244].

Populations living in the area of Mediterranean Sea showed a decrease incidence of cancer compared with those living in regions of North Europe or US, this has been attributed to healthier dietary habits [245]. In the past decade, several reports indicated a protective role of MD towards neoplastic diseases. In particular, a meta-analysis study by Sofi and colleagues reported that MD is responsible of 6% reduction of cancer death/incidence [246]. An updated study, done on a very large cohort (335,873 subjects of the European Prospective Investigation Into Cancer and Nutrition), reported a lower cancer risk in subjects following MD [247]. Nowadays, several epidemiological studies focused on the association of MD and specific type of cancers (breast and colorectal cancers).

The worst cancer in women is breast cancer, which accounts for over 25% of all female cancers. Estrogen has a key role in the pathogenesis and progression of this cancer. The MeDiet study investigated the effect of the MD on the levels of endogenous estrogens in healthy postmenopausal women [248]. The women enrolled were divided into two different groups: the first group was treated with the MD for six months, while the control group continued the current diet. Urine samples were examined in order to assess the levels of estrogen, which reflect its intra-tissutal content [249] The majority of urinary estrogen is represented by hydroxy and methoxy derivatives of estradiol (E2) and estriol (E3). The study revealed that the group receiving MD had reduced levels of hydroxy and keto derivatives of E2 and E3, known as 2-hydroxy E2 (2OHE2), 17EpiE3 and 16KetoE2, respectively 80, 70 and 27% after six months, while they were unchanged in the control group. The study confirmed the protective role of the MD on cancer development, acting on estrogen metabolism.

Regarding to the colon rectal cancer, a recent study [250] showed that this neoplasia is the third most common in US. The authors associated colon rectal cancer risk, MD Score (MDS) and the Healthy Eating Index (HEI) furthermore; they define a novel Dietary Inflammatory Index (DII). Comparing different published studies (US and European patients), the authors established that higher MDSs were associated with lower colorectal cancer risk (8-54%), as well as higher HEI scores were associated with lower colorectal cancer risk (20-56%). On the other hands, pro-inflammatory diets were associated with a 12-65% higher colorectal cancer risk compared with more anti-inflammatory diets in studies that used the DII [250]. The latter result is very interesting as unresolved inflammation, unrelated to infections, observed in obesity, can contribute to carcinogenesis as observed in Barrett's metaplasia, chronic pancreatitis or esophagitis [251].

The MD contributes to the prevention of colorectal cancer through a high intake of fiber. Barera et al. have highlighted the nutraceutical effects of β-glucans, which seem to reduce low-density lipoprotein cholesterol (LDL), IL-6 and advanced glycation end-product (AGE) levels. They are also linked to colon cancer prevention [252].

The MD is able to reduce gastric cancer's incidence and mortality in the South areas of selected Mediterranean country, such as France, Greece and Italy when compared with the North areas of the same countries. Higher adherence to MD lower up of 20% the incidence of all gastric cancers [253–255].

The beneficial role of MD has also been confirmed in a large case-control study in Italy. The authors found that an enhanced adherence to MD (high consumption of fruit, vegetables and legumes) reduced risk of both oral cavity and pharynx cancer and larynx cancer [256].

Head and neck cancers are currently the sixth cause of death in the world; however, as confirmed by Bosetti et al., a greater adherence to the MD reduces the risk of oral, laryngeal and pharyngeal cancer (by 23 and 29%, respectively) [257].

Jacobs et al. have suggested that consumption of whole grain (bran, germ, endosperm) ≥ 4 times / week reduces the risk of cancer by 40% compared to controls, while the Continuous Update Project has determined that the intake of non-starchy vegetables and fruits lowers the risk of mouth, pharynx, larynx, esophagus and stomach cancers [258, 259].

On the other hand, the intake of refined cereal grains (bread, pasta or rice) increase the risk of upper digestive tract, stomach, colorectal, breast and thyroid cancer [260].

Another pivotal food of the MD is pulses. Thanks to their content in tannins, saponins, protease inhibitor and phytic acid, legumes play an important anti-cancer role [261].

Even the consumption of nuts was associated to the reduction of cancer's risk, in particular for those of the digestive tract. The protective role of nuts comes from their content of ellagic acid, anacardic acids, genistein, resveratrol and phytic acid [262]. The consumption of peanut products ≥ 2 / week was associated with 58% reduction of colorectal cancer risk in Taiwan women [263].

According to IARC, ethanol was positively correlated to cancers (e.g. mouth, pharynx, larynx, oesophagus, colorectal in men, breast in pre and post-menopause). The Continuous Update Project also confirmed the carcinogen effect of alcohol in liver and colorectal cancers [264]. However, the studies on MD show that a moderately intake of red wine in pre-menopause women reduced breast cancer risk, inhibiting the conversion of androgens to estrogens, catalyzing by aromatase [113, 265] This effect is due to polyphenols content of red wine, including flavonoids (anthocyanins and flavan-3-ols) and non-flavonoids (resveratrol, cinnamates and gallic acid [266].

**Figure 2:**
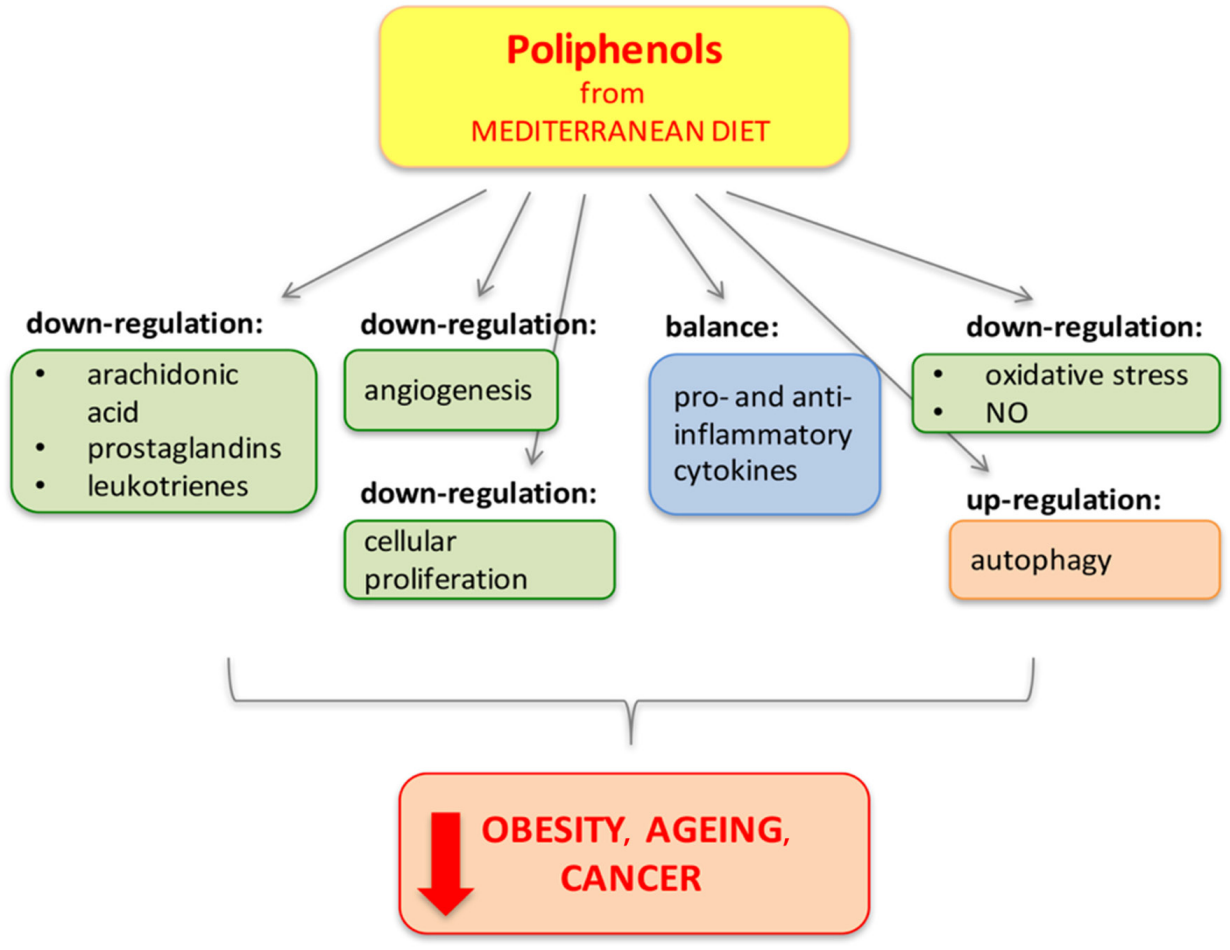
Polyphenols from Mediterranean Diet Polyphenols protect and reduce inflammation by different pathways (through mechanisms of down-regulation, balance and up-regulation) preventing obesity, cancer and age-related diseases, in which inflammation has an important pathological role [240].

The role of main components of the MD in the prevention of several cancers type has been recently reviewed in several works [245, 267, 268, 269] (Figure 2).

## MEDITERRANEAN DIET AND LONGEVITY

Cellular aging consists in a loss of cellular physiological functions, which occurs gradually over time. The most important biological marker of this process is represented by telomere shortening that affects life expectancy and increases the individual susceptibility to the development of chronic diseases [270, 271]. Eating habits and metabolic factors (particularly an increased visceral adipose tissue and circulating glucose levels) cause a faster shortening of telomeres and a reduction telomerase activity, suggesting a key role of the environment in cellular senescence [272]. Several survival-population studies have demonstrated that a diet rich in fruits, vegetables, fish and low in fat foods, is correlated to lower incidence of chronic diseases and higher survival [273, 274]. In particular, a greater adherence to MD (expressed as higher MDS) was related to longer leukocyte telomere length, higher telomerase activity and lower plasmatic level of pro-inflammatory cytokines (such as IL-6 and TNF-α) and a reduction of oxidative stress [275].

Tiainen et al. reported that fruit and vegetables consumption was positively linked to leukocyte telomere length in women aged 57-70 years, but not in men with the same age [277].

The MDS, together with the Mediterranean Adequacy Index (MDI) and the Healthy Diet Indicator (HDI), were studied during the Healthy Ageing: a European longitudinal study (HALE) that included men and women from ten European countries. The subjects were followed for a 10 years-time observation. The study has confirmed the protective role of MD for elderly subjects with and without baseline chronic diseases [277]. A recent prospective study involving 4676 healthy women has demonstrated the relationship between adherence to the MD and telomere length in cells, supporting the concept of the benefits of MD to promote health status and longevity. Any association was noted between the individual MD components and longevity, but was supposed a synergy effect among the nutrients rich foods included in MD [278]. In general, subjects who adhere to the traditional MD have a longer life - span [275]. Another study confirmed that higher adherence to MD in older adults in the United-States and Israel is linked to better cognitive and physical functions. In fact, in this subjects were detected a faster walking speed and fewer disabilities [279]. The PREDIMED trial reports how the MD supplemented with olive oil (1 l/wk) or mixed nuts (30 g/d) is linked to lower cognitive decline, evaluated through standardized neuropsychological tests in older populations [280]. In a Greek population cohort study, Trichopoulou et coworkers demonstrated for the first time that higher degree of adherence to MD was correlated with a reduction in total mortality; an increase of two point of MDS was associated with a diminution of 25% mortality of all-causes. This reduction was more evident for CVD respect to cancer [281]. A subsequent meta-analysis of prospective studies confirmed a significant protective role of MD against major chronic degenerative disease. In fact, an increase of two-points of MDS induced 8% reduction of death from any causes, a 10% reduction from death and/or incidence of cardio and cerebro-vascular disease and 13% reduction of neuro-degenerative disease [246].

Another paper has compared the effects of three different diets, following for four weeks, on cellular senescence: saturated fatty acids diet, a low-fat/high carbohydrate diet and MD enriched in MUFA. The study concluded that MD prevents endothelial-cells aging and decreased intracellular oxidative stress, cellular apoptosis and telomere shortening. Moreover, the MD was associated with an improvement in the regenerative capacity of endothelium, in comparison with other diets [282, 283]. In order to explain the role of MD on telomere lengths could be considered the protective action of olive oil (OO), in particular virgin olive oil polyphenols, on mitochondrial and nuclear DNA against oxidative stress. The OO induces important epigenetic changes through its MUFA content and polyphenolic compounds; in fact, OO can prevent and palliate some aging-associated disease such as diabetes [284].

The OO protective effect on aging is confirmed by another study that has evaluated the bioactivity of OO compounds on multipotential mesenchymal stem cell (MSC) progenitor differentiation. In fact, osteoblasts and adipocytes derive from the same MSC. Santiago-Mora et al. have demonstrated that oleuropein, one of the most important OO compounds, enhances osteoblastogenesis and inhibits the main adipogenesis regulators, as PPAR-γ2, lowering the incidence of osteoporosis [285]. Furthermore, the phenolic compound oleocantale has the same anti-inflammatory effects of ibuprofen, the most common non-steroidal anti-inflammatory. In fact the OO intake decreases inflammation and consequently aging [286].

The benefits of MD are evident in the prevention and slowing the progression of chronic degenerative diseases. A meta-analysis highlighted that both men and women who nearest eating habits to MD have a reduced risk of about 10-20% to die for CVD, cancer and other any causes. The MD is significantly inversely associated with both systolic and diastolic BP. A prospective study on 41358 Spanish adult subjects, during 6.5 years of follow-up, evaluated the possible relationship between dietary factors and total mortality. The authors showed that high intakes of fresh fruit and vegetables are associated with decreased mortality, probably related to the high concentration on vitamin C, provitamin A, carotenoids and lycopene. Antioxidant capacity of vitamin C and provitamin A partly explain this effect on mortality [287]. The MD, poor in refined sugar and animal proteins, reduces the activity of mTOR and insulin/IGF-1 pathways, lowering the risk of age-related diseases, promoting successful aging and longevity [288].

To confirm the protective role of the MD, the Lion Diet Heart Study has shown a reduction in secondary cardiac events, including death and non-fatal myocardial infarction, after a first myocardial infarction. This protective effect remains at least four years after the first heart attack, independent of main cardiovascular risk factors, such as hypertension and dyslipidemia [289–291].

The benefit effect of MD has confirmed also on elderly non-smoking people. In this study 161 nonsmoking individuals, aged 65-90, were separated into two different groups: the first were 65-80 years, the latter > 80 years. The study has demonstrated that the total diet score, based on 8 main components of MD, is positivity associated to higher longevity in patients less 80 years, but not in those greater than 80. The authors have noted also that higher albumin concentration is linked to lower mortality [292].

The MD includes a high significantly quantity of foods rich in antioxidant compounds, which can help explain its many benefits. The extra virgin olive oil, vegetables, fresh fruits, nuts, wine and fish, contain molecules with anti-oxidants and anti-inflammatory properties, as monounsaturated fatty acids, fatty acids omega 3, polyphenols, flavonoids, phytosterols, vitamins, antioxidants, minerals and micronutrients. These factors act on longevity and slow the development of chronic diseases associated with aging [293].

MD effect on telomere length makes itself a powerful anti-aging tool.

## CONCLUSION

MetS is characterized by a cluster of metabolic alterations that can conduce to NTCDs. Several epidemiological, clinical and experimental studies suggested that both the increased quantity and the dysfunctional quality of AT, called “adiposopathy”, cause the onset of chronic metabolic diseases and increases cancer incidence. The diet and lifestyle are gaining an increasingly important role, both for the treatment and the prevention of NTCDs. Early population diet-therapy intervention and the study of genetic polymorphism allow the prevention and slow the progression of chronic degenerative diseases (Table 2 and 3, Figure 2). In 2010, the UNESCO proclaimed the MD as “World Cultural Heritage”. This diet represents a behavioural model, a “way of life”, that can ensure longer life expectancy and improve quality of life itself.

## Abbreviations

a MED: alternate Mediterranean Diet Score
ACSL1: long-chain acyl CoA synthetase 1
AHEI-2010: the Alternative HEI-2010
AI: Atherogenic Index
AT: Adipose tissue
ATGL: fatty triglyceride lipase
BAT: brown adipose tissue
BCM: body cell mass
BMD: bone mineral density
BP: blood pressure
CAPN10: Calpain 10
CHD: cardiac heart disease
CKD: chronic kidney disease
COX: ciclo-oxigenase
CSF1: colony stimulating factor-1
CSI: Cholesterol/Saturated Fat Index
CVD: cardiovascular diseases
DASH: Dietary Approaches to Stop Hypertension
DHA: docosahexaenoic acid
DII: Dietary Inflammatory Index
e-GFR: estimated glomerular filtration rate
EAT: Epicardial Adipose Tissue
EPA: Eicosapentaenoic acids
FFA: free fatty acids
FTO: fat mass and obesity-associated
GISSI: Gruppo Italiano per lo Studio della Sopravvivenza nell’Infarto Miocardico
GST: glutathione S-transferase
GWAS: Genome-wide association studies
HALE: Healthy Ageing: a European longitudinal study
Hcy: homocysteine
HDI: Healthy Diet Indicator
HEI-2010: Healthy Eating Index-2010
HRNMD: Healthy Reference National Mediterranean Diet
hs-CRP: high-sensitive C- Reactive Protein
HSL: hormone-sensitive lipase
IDF: International Diabetes Federation
IL-1: interleukin-1
IL-6: interleukin-6
IMAT: intermuscular adipose tissue
IMOD: Italian Mediterranean Organic Diet
iNOS: inducible nitric oxide synthase
IR: insulin resistance
KANWU: Kopio, Aahhus, Naples, Wollongon and Uppsala
LEP: leptin
LEPR: leptin receptor
MAI: Mediterranean Adequacy Index
MC4R: melanocortin-4 receptor
MCP-1: monocyte-chemotactic protein-1
MD: Mediterranean Diet
MDA: malondialdehyde
MDI: Mediterranean Adequacy Index
MDS: Mediterranean Diet Score
MetS: Metabolic Syndrome
MHMD: moderately hypocaloric Mediterranean Diet
MHO: Metabolically Healthy Obese
MONW: Metabolically Obese Normal Weight
MTHFR: methylenetetrahydrofolate reductase
MUFObes: MUFA in Obesity
MUO: Metabolically Unhealthy Obese
NCEP ATP III: National Cholesterol Education Program's Adult Treatment Panel III report
NO: nitric oxide
NOW: Normal Weight Obese
NTCDs: Non-transmissible Chronic Diseases
OO: olive oil
PAI-1: pro-coagulant substances
PBF: body fat percentage
POMC: pro-opiomelanocortin
PPARγ: peroxisome proliferator-activated receptor γ
PREDIMED: PREvenciòn con DIeta MEDiterrànea
PUFA: polyunsaturated fatty acids
ROS: Reactive Oxygen Species
RS: renal sinus
SAT: subcutaneous adipose tissue
SFA: saturated fatty acid
SNPs: single nucleotides polymorphisms
SOD: superoxide-dismutase
t-MED: MDS with fish
T2DM: type 2 diabetes mellitus
TAC: Total antioxidant capacity
TCF7L2: transcription factor 7-like 2
TI: Thrombogenic Index
TNF-α: tumor necrosis factor alpha
UAE: urinary albumin excretion
UNESCO: United Nations Educational, Scientific and Cultural Organization
VAT: visceral adipose tissue
VCAM1: vascular cell adhesion-1
VDR: vitamin D receptor
WAT: white adipose tissue
